# Supplementary material for: Soil and Vegetation Development on Coal-Waste Dump in Southern Poland
Source: Int J Environ Res Public Health. 2022 Jul 27;19(15):9167. doi: 10.3390/ijerph19159167 (PMC9368154; doi:10.3390/ijerph19159167)

Table S2. Correlation analysis of physico-chemical properties of soils (Spearman rank correlation coefficient).

Variable	pH H_2O	pH KCl	Loss ignition	Corg.	Nt	C/N	Mg avail.	P avail.	Pt	K avail.	Hh
**pH H_2O**	1	0.971***	-0.154	-0.089	-0.455	0.182	0.208	0.078	0.593*	0.630*	-0.614*
**pH KCl**	0.971***	1	-0.127	-0.069	-0.520*	0.224	0.304	0.138	0.651**	0.718**	-0.648**
**Loss ignition**	-0.154	-0.127	1	0.860***	-0.182	0.685**	-0.100	-0.493	-0.171	-0.257	-0.237
**Corg.**	-0.089	-0.069	0.860***	1	-0.382	0.857**	0.096	-0.327	-0.060	-0.225	-0.394
**Nt**	-0.455	-0.520*	-0.182	-0.382	1	-0.746**	-0.521*	0.175	-0.110	-0.380	0.620*
**C/N**	0.182	0.224	0.685**	0.857***	-0.746**	1	0.232	-0.373	-0.010	0.094	-0.672**
**Mg avail.**	0.208	0.304	-0.1	0.096	-0.521*	0.232	1	0.110	0.250	0.436	-0.082
**P avail.**	0.078	0.138	-0.493	-0.327	0.175	-0.373	0.110	1	0.602*	0.156	0.222
**Pt**	0.593*	0.651**	-0.171	-0.060	-0.110	-0.010	0.250	0.602*	1	0.529	-0.285
**K avail.**	0.630*	0.718**	-0.257	-0.225	-0.380	0.094	0.436	0.156	0.529*	1	-0.502
**Hh**	-0.614*	-0.648**	-0.237	-0.394	0.620*	-0.672**	-0.082	0.222	-0.285	-0.502	1

Asterisks indicate a correlations are significant at *$P < 0.05$; **$P < 0.01$; ***$P < 0.001$).

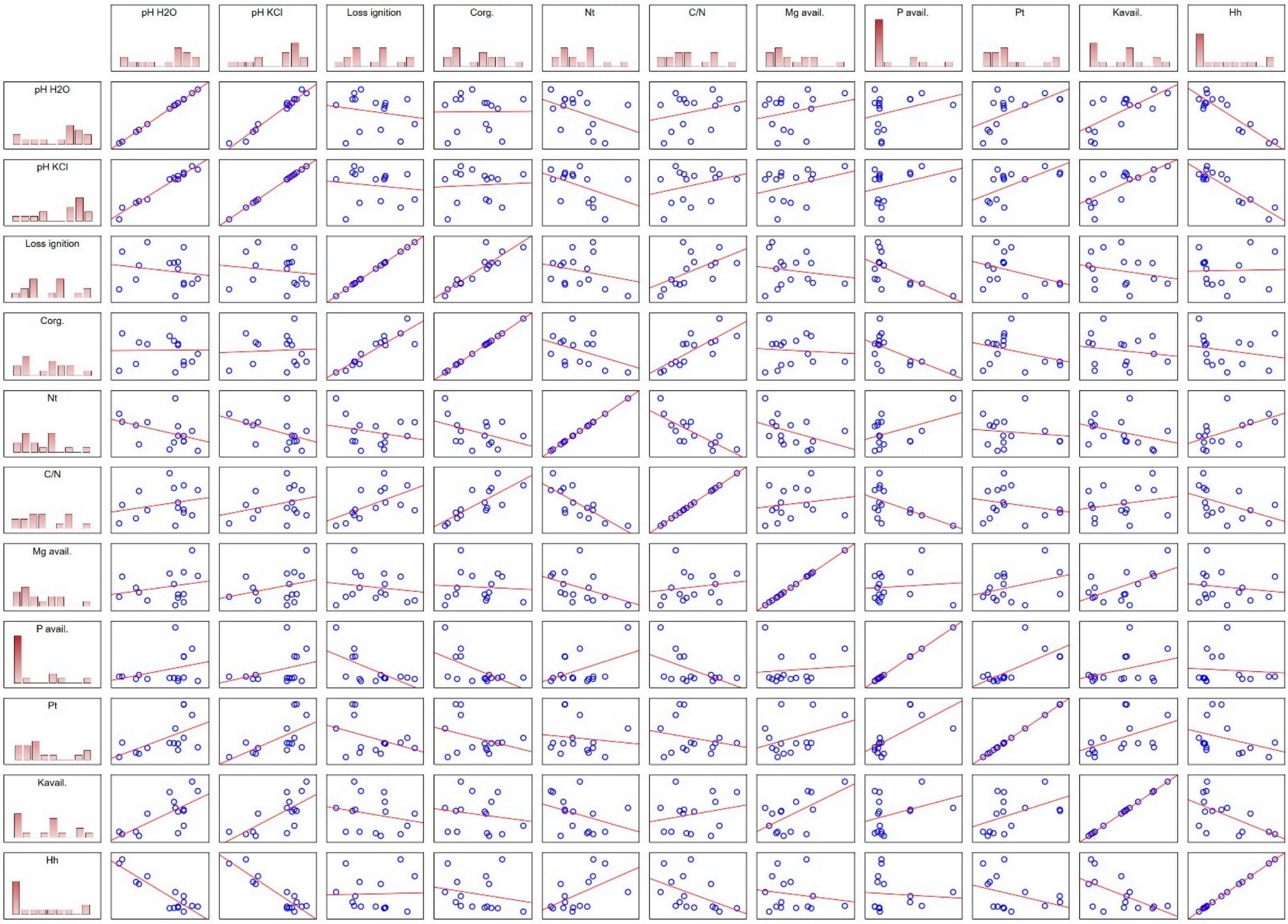

Supplement: Supplementary file 1 [file ijerph-19-09167-s001.zip › Table S2.pdf]
