# Supplementary material for: Soil and Vegetation Development on Coal-Waste Dump in Southern Poland
Source: Int J Environ Res Public Health. 2022 Jul 27;19(15):9167. doi: 10.3390/ijerph19159167 (PMC9368154; doi:10.3390/ijerph19159167)
Supplement: Supplementary file 1 [file ijerph-19-09167-s001.zip › Table S3.pdf]

Table S3. The main ogranic compound in analized soils.

		Profile 3			Profil 2			Proil 1		Profil 4				
		Horizons												
		A	ACq	Cq	A	ACq	Cq	C1	C1	OC				
	m/z													
n-Undecane	71	0.12	-	-	0.37	0.43	0.24	0.44	0.43	-	0.12	-	0.22	0.89
n-Dodecane	71	0.32	0.16	0.14	0.11	0.59	0.29	0.34	0.78	0.17	0.08	0.06	0.19	0.12
n-Tridecane	71	1.43	1.28	0.92	0.94	3.03	2.22	2.58	2.96	0.05	0.30	0.24	0.76	0.20
n-Tetradecane	71	2.83	3.23	2.63	2.93	6.10	5.19	5.87	5.84	0.42	1.71	1.16	2.00	0.72
n-Pentadecane	71	3.57	4.73	3.87	5.63	6.86	6.37	7.07	6.82	2.07	2.63	1.38	2.44	1.87
n-Hexadecane	71	3.53	5.23	4.55	6.11	7.78	6.82	7.80	7.79	4.26	2.76	1.89	2.40	1.63
n-Heptadecane	71	4.08	6.26	5.40	6.94	7.47	6.97	7.83	8.11	5.61	7.07	6.61	4.03	0.46
Pristane	71	15.95	13.84	16.79	10.91	10.61	11.00	8.34	6.48	7.64	7.94	3.86	4.91	10.74
n-Octadecane	71	4.24	6.23	5.36	7.29	6.89	7.18	7.27	7.78	7.97	3.03	1.72	1.72	4.21
Phytane	71	1.73	1.99	2.07	1.84	1.61	1.64	1.78	1.50	6.42	1.33	0.75	0.79	1.70
n-Nonadecane	71	4.14	6.46	5.73	6.85	6.29	7.44	6.14	7.70	1.75	2.68	1.43	1.46	1.69
n-Eicosane	71	3.90	5.74	5.43	6.22	5.38	7.42	5.53	6.73	6.01	2.37	1.29	1.52	1.67
n-Heneicosane	71	4.48	6.10	5.37	5.99	5.23	6.08	5.23	5.57	5.93	2.77	1.11	1.68	1.60
n-Docosane	71	3.84	5.02	4.46	4.77	4.82	4.89	4.72	4.95	5.62	2.12	1.15	1.33	1.78
n-Tricosane	71	4.10	4.77	4.55	4.43	4.68	4.36	4.60	4.58	4.64	2.16	1.23	1.76	1.30
n-Tetracosane	71	3.74	4.73	4.12	3.90	4.37	3.80	4.13	4.15	4.65	2.04	1.04	1.25	1.05
n-Pentacosane	71	3.80	4.25	4.31	3.77	3.88	3.36	3.61	3.31	4.48	2.43	1.90	2.38	1.42
n-Hexacosane	71	3.31	3.69	3.51	3.03	3.15	2.82	3.09	2.85	4.30	1.57	0.95	1.21	0.96
n-Heptacosane	71	4.64	3.84	4.27	3.58	2.88	2.64	2.87	2.49	3.39	3.58	2.70	4.78	2.38
n-Octacosane	71	2.67	2.57	2.62	2.04	2.15	1.83	2.25	1.75	4.21	1.00	0.72	0.94	0.82
n-Nonacosane	71	4.55	2.46	3.14	2.42	1.76	1.65	1.94	1.51	2.60	3.72	3.52	4.92	2.99
n-Tricontane	71	1.49	1.20	1.40	1.35	1.18	1.14	1.35	0.92	3.39	0.79	0.61	0.88	0.67
n-Hentriacontane	71	2.22	1.20	1.52	1.52	0.85	0.81	1.09	0.86	1.45	2.11	3.57	3.40	3.88
n-Dotriacontane	71	0.66	0.91	0.59	0.70	0.55	0.40	0.63	0.50	1.72	0.42	0.26	1.10	1.38
n-Tritriacontane	71	0.69	0.57	0.62	0.60	0.33	0.41	0.55	0.37	0.82	0.66	0.63	-	-
n-Tetratriacontane	71	-	-	-	-	-	-	-	-	0.74	-	-	-	-
n-pentanoic acid	60	0.26	0.08	0.15	0.07	0.04	0.07	0.10	0.05	0.11	0.09	0.33	0.11	0.14
n-Hexanoic acid	60	0.53	0.24	0.32	0.37	0.18	0.35	0.41	0.51	0.43	0.35	0.15	0.44	0.34
n-Heptanoic acid	60	0.28	0.14	0.18	0.13	0.05	0.13	0.14	0.14	0.21	0.18	0.29	0.20	0.22
n-Octanoic acid	60	0.14	0.19	0.20	0.23	0.10	0.18	0.21	0.24	0.31	0.39	0.23	0.41	0.35
n-Nonanoic acid	60	0.30	0.20	0.20	0.21	0.10	0.16	0.18	0.31	0.32	0.45	0.19	0.25	0.18
n-Decanoic acid	60	0.21	0.14	0.15	0.13	0.05	0.08	0.12	0.12	0.19	0.23	0.07	1.65	0.16
n-Undecanoic acid	60	0.14	0.08	0.09	0.07	0.02	0.05	0.04	0.03	0.14	0.06	0.29	1.51	0.53
n-Dodecanoic acid	60	0.17	0.09	0.14	0.10	0.03	0.09	0.05	0.08	0.15	0.25	0.04	0.24	0.12
n-Tridecanoic acid	60	0.11	0.08	0.09	0.06	0.02	0.04	0.03	0.04	0.08	0.08	0.43	0.03	0.07
n-Tetradecanoic acid	60	0.19	0.09	0.15	0.11	0.03	0.06	0.03	0.05	0.15	0.34	0.10	0.40	0.49
n-Pentadecanoic acid	60	0.13	0.03	0.08	0.05	0.02	0.04	0.02	0.02	0.07	0.10	2.72	0.17	0.64
n-Hexadecanoic acid	60	0.75	0.17	0.45	0.56	0.34	0.65	0.17	0.26	0.79	1.80	0.06	3.87	4.09
n-Heptadecanoic acid	60	0.09	0.02	0.03	0.03	0.01	0.04	0.02	0.03	0.03	0.04	0.62	0.10	0.43
n-Octadecanoic acid	60	0.13	-	0.05	0.05	0.02	0.02	0.01	0.06	0.11	0.31	0.12	0.93	-
n-Nonadecanoic acid	60	-	-	-	0.04	0.05	0.03	0.04	-	-	0.14	-	0.17	-
n-Eicosanoic acid	60	-	-	-	0.05	-	-	-	-	-	-	-	-	-

δ-Tocopherol	151	0.25	-	0.18	-	-	-	-	-	-	0.65	0.57	0.48	0.85
γ-Tocopherol	165	1.22	-	0.60	0.38	-	-	-	-	0.72	3.88	4.99	3.24	3.31
Cholestanol	215	-	-	-	-	-	-	-	-	-	0.53	1.47	0.96	0.91
5β-Cholestan-3β-ol (coprostanol)	215	-	-	-	-	-	-	-	-	-	1.34	8.70	6.78	4.41
Cholest-5-en-3β-ol (cholesterol)	231	-	-	-	-	-	-	-	-	-	0.83	3.66	2.90	2.21
Cholestan-3-one	231	-	-	-	-	-	-	-	-	-	0.90	1.40	1.03	1.15
Cholest-4-en-3-one	124	-	-	-	-	-	-	-	-	-	-	-	0.47	-
Stigmastanol	218	-	-	-	-	-	-	-	-	-	0.31	0.59	0.25	0.37
Stigmastan-3β-ol	215	-	-	-	-	-	-	-	-	-	0.60	2.09	1.22	1.27
Stigmast-5-en-3β-ol (β-sitosterol)	231	0.18	0.15	-	-	-	-	-	-	0.24	0.79	1.56	0.92	1.16
5α-stigmastan-3-one	231	0.34	0.13	-	-	-	-	-	-	-	0.73	0.83	0.54	0.72
Stigmasta-3,5-dien-7-one	174	0.34	-	-	-	-	-	-	-	0.08	0.32	0.27	0.17	0.47
Stigmast-4-en-3-one (Sitostenone)	124	0.65	-	0.36	0.34	-	-	-	-	0.29	0.58	0.65	0.39	0.72
5α-Ergostan-3β-ol (ergostanol)	215	-	-	-	-	-	-	-	-	-	0.16	0.81	0.45	0.46
Styrene (styrol)	104	1.06	0.04	0.26	-	-	-	-	-	0.02	2.10	0.76	1.08	0.94
2-Methoxyphenol	124	0.04	-	-	-	-	-	-	-	-	-	-	-	-
Methyl olean-12-en-3-oxo-28-oate (Methyl oleanonate)	189	0.75	-	-	-	-	-	-	-	-	-	-	-	-
D-Friedoolean-14-en-3-one	300	0.37	-	0.12	-	-	-	-	-	-	0.26	0.08	-	-
3-Ethyl-4-methyl-1H-pyrrole-2,5-dione (Methylethylmaleimide)	139	0.31	0.46	0.41	0.13	0.09	0.12	0.22	0.15	0.20	0.24	0.15	0.20	0.21
β-amyrone	218	0.17	-	0.15	0.33	-	-	-	-	-	0.42	0.78	0.53	-
β-amyrine	218	0.24	-	0.13	0.40	-	-	-	-	-	0.63	0.99	0.57	-
α-amyrine	218	0.37	-	0.16	0.37	-	-	-	-	-	0.55	0.96	0.60	-
Urs-12-en-3β-ol, acetate (α-Amyrin acetate)	218	0.12	-	-	0.20	-	-	-	-	-	0.07	0.27	0.19	-
3β-cholestane-3-thiol	249	-	-	-	-	-	-	-	-	-	8.35	5.93	3.44	5.34
1,2,4-Trithiolane	124	0.08	0.09	0.11	0.06	-	0.05	0.04	-	-	0.13	0.08	0.11	0.12
Benzenemethanethiol	124	-	-	-	-	-	-	-	-	-	-	0.04	-	-
3-Methyl-2-(3,7,11-trimethyldodecyl)thiophene	111	-	-	-	-	-	-	-	-	-	1.78	1.98	2.40	0.64
3-*n*-Hexadecylthiophene	98	-	-	-	-	-	-	-	-	-	0.85	0.99	1.11	0.27
4-Methylbenzenemethanethiol	105	0.26	-	0.07	-	-	-	-	-	0.81	-	-	0.49	-
benzoic acid	77	0.37	-	-	-	-	-	-	-	-	-	0.09	-	-
2-hydroxybenzaldehyde (salicylaldehyde)	122	0.40	0.09	0.20	0.02	-	0.13	0.08	-	-	-	-	-	-
Methyl benzoate	105	0.24	0.37	0.18	0.27	-	0.10	0.18	-	-	-	-	-	1.87
4-Hydroxy-3-methoxybenzaldehyde (vanillin)	136	0.43	0.14	0.24	0.06	-	0.16	0.18	0.19	0.09	0.02	0.01	0.06	-
3-Hydroxy-4-methylbenzaldehyde	136	0.50	0.17	0.33	0.11	-	0.17	0.27	0.24	0.25	0.05	0.03	0.08	-
2-Hydroxy-5-methylbenzaldehyde	136	0.33	0.10	0.22	0.07	-	0.08	0.10	0.11	0.13	0.03	0.03	0.06	-
2-Hydroxy-3-methylbenzaldehyde	136	0.15	0.05	0.09	0.05	-	0.05	0.11	0.18	0.16	0.03	0.02	0.02	-
7,11,15-Trimethyl-3-methylenehexadec-1-ene (Neophytadiene)	123	-	-	-	-	-	-	-	-	0.83	1.78	2.17	0.00	3.59
3,7,11,15-Tetramethylhexadec-2-ene (Phytene-2)	123	-	-	-	-	-	-	-	-	0.11	0.14	0.12	0.00	0.23
Trans-3,7,11,15-Tetramethyl-1,3-Hexadecadiene (Phytadiene 1)	55	-	-	-	-	-	-	-	-	1.09	2.54	3.06	1.08	5.56
phytadiene	123	-	-	-	-	-	-	-	-	0.15	0.24	0.22	0.61	0.74
phytol	71	-	-	-	-	-	-	-	-	0.33	0.88	1.18	0.66	2.24
Tetradecan-1-ol	55	-	-	-	-	-	-	-	-	-	0.53	0.54	0.64	0.54
10-Dodecen-1-ol	55	-	-	-	-	-	-	-	-	-	0.27	0.34	0.30	1.14
8-Heptadecene	55	-	-	-	-	-	-	-	-	-	1.69	2.06	1.20	2.58
1-octadecene	55	-	-	-	-	-	-	-	-	-	0.60	0.34	1.63	-
1-Tricosene	55	-	-	-	-	-	-	-	-	-	-	0.37	-	-
1-Tetracosene	55	-	-	-	-	-	-	-	-	0.82	1.23	1.13	1.75	0.75
Indole	117	-	-	-	-	-	-	-	-	-	0.32	0.44	0.75	0.13
Indene	115	0.88	0.04	0.25	0.21	-	-	-	-	0.04	-	1.16	1.53	1.17
Benzaldehyde	77	0.22	0.07	0.11	0.04	-	0.08	0.02	0.21	0.07	0.09	-	-	-
Acetophenone	77	0.18	0.08	0.12	0.07	-	0.14	0.17	0.25	0.18	-	-	-	-
CPI(n-C_{24}-C_{34})		1.65	1.21	1.42	1.37	1.11	1.16	1.13	1.13	0.79	2.72	4.13	3.31	2.48
CPI(n-C_{25}-C_{31}) alkanes		1.63	1.21	1.37	1.34	1.08	1.10	1.07	1.10	0.75	2.84	3.92	4.15	3.27

Σshort chain/Σlong chain	1.15	1.67	1.43	1.98	2.36	2.63	2.33	2.81	1.10	1.35	1.05	0.87	1.00
Σalkanes/Σalkanoic acids	19.95	52.29	32.62	36.05	83.31	42.33	54.95	45.87	24.62	10.04	6.24	4.03	4.34

CPI(n-C_{24} - n-C_{34}) alkanes = $(((C_{25} + C_{27} + C_{29} + C_{31} + C_{33})/(C_{24} + C_{26} + C_{28} + C_{30} + C_{32})) + ((C_{25} + C_{27} + C_{29} + C_{31} + C_{33}) + (C_{26} + C_{28} + C_{30} + C_{32} + C_{34}))) * 0.5$ [1],

CPI(n-C_{25}- n-C_{31}) alkanes=((C25+C27+C29)+(C27+C29+C31))/(2 ∗ (C26+C28+C30)) [2]

Σshort chain(n-C_{11}-n-C_{22}) / Σlong chain(n-C_{23}- n-C_{34}) alkanes; m/z = 71 [3]

References

1. Bray, E.E., Evans, E.D., 1961. Distribution of n-parafins as a clue to recognition of source beds. Geochim. Cosmochim. Acta 22, 2–15.
2. Kotarba, M.J., Solarski, W., Stecko, Z., 1994. Nowa metoda obliczeń wskaźnika CPI i wykorzystanie dystrybucji n-alkanów i izoprenoidów prospekcji naftowej – instrukcja metodyczna analiz z zakresu geochemii organicznej dla potrzeb poszukiwań naftowych. Materiał. wewnętrzne AGH 82–91 (in Polish).
3. Tissot, B., Welte, D.H., 1984. Petroleum Formation and Occurrence. A New Approach to Oil and Gas Exploration. 2nd edn. Springer – Verlag, Berlin, Heidelberg, New York, p 699.
