# Supplementary material for: Soil and Vegetation Development on Coal-Waste Dump in Southern Poland
Source: Int J Environ Res Public Health. 2022 Jul 27;19(15):9167. doi: 10.3390/ijerph19159167 (PMC9368154; doi:10.3390/ijerph19159167)
Supplement: Supplementary file 1 [file ijerph-19-09167-s001.zip › Table S4.pdf]

Table S4. Correlation analysis of the content of major elements in plant material (Spearman rank correlation coefficient).

Variable	Ca	K	Na	Mg	P	Fe	S	Al
**Ca**	1	0.095	-0.395	0.238	-0.714*	-0.380	-0.143	-0.409
**K**	0.095	1	-0.419	0.309	0.166	-0.380	0.107	-0.327
**Na**	-0.395	-0.419	1	0.179	0.059	0.826*	0.174	0.589
**Mg**	0.238	0.309	0.179	1	-0.142	-0.07	0.011	-0.109
**P**	-0.714*	0.166	0.059	-0.142	1	-0.02	0.275	0.218
**Fe**	-0.380	-0.380	0.826*	-0.071	-0.02	1	0.419	0.872**
**S**	-0.143	0.107	0.174	0.011	0.275	0.419	1	0.452
**Al**	-0.409	-0.327	0.589	-0.109	0.218	0.872**	0.452	1

Asterisks indicate a correlations are significant at *$P < 0.05$; **$P < 0.01$; ***$P < 0.001$).
