# Supplementary material for: Soil and Vegetation Development on Coal-Waste Dump in Southern Poland
Source: Int J Environ Res Public Health. 2022 Jul 27;19(15):9167. doi: 10.3390/ijerph19159167 (PMC9368154; doi:10.3390/ijerph19159167)
Supplement: Supplementary file 1 [file ijerph-19-09167-s001.zip › Table S5.pdf]

Table S5. Correlation analysis of major element content in soil (Spearman rank correlation coefficient).

Variable	Ca	K	Na	Mg	P	Fe	S	Al
**Ca**	1	-0.513	0.728*	0.790*	0.682	-0.179	0.467	-0.179
**K**	-0.513	1	-0.132	-0.740*	-0.280	0.446	0.140	0.038
**Na**	0.728*	-0.132	1	0.312	0.299	0.156	0.847**	-0.665
**Mg**	0.790*	-0.740*	0.312	1	0.476	-0.190	-0.095	0.238
**P**	0.682	-0.280	0.299	0.476	1	-0.071	0.404	-0.142
**Fe**	-0.179	0.446	0.156	-0.190	-0.071	1	0.261	-0.214
**S**	0.467	0.140	0.847**	-0.095	0.404	0.261	1	-0.857**
**Al**	-0.179	0.038	-0.665	0.238	-0.142	-0.214	-0.857**	1

Asterisks indicate a correlations are significant at *$P < 0.05$; **$P < 0.01$; ***$P < 0.001$).
