# Supplementary material for: Soil and Vegetation Development on Coal-Waste Dump in Southern Poland
Source: Int J Environ Res Public Health. 2022 Jul 27;19(15):9167. doi: 10.3390/ijerph19159167 (PMC9368154; doi:10.3390/ijerph19159167)

Table S6. Correlation analysis of heavy metal content in plant material (Spearman rank correlation coefficient).

Variable	Zn	Pb	Cd	Co	Ni	Hg	Cu	As	Sr	Cr
**Zn**	1	0.500	0.452	0.547	0.428	0.694	0.785***	0.327	0.119	0.850**
**Pb**	0.500	1	0.880**	0.523	0.428	0.670	0.619	0.545	0.642	0.706
**Cd**	0.452	0.880*	1	0.261	0.261	0.742*	0.571	0.709*	0.333	0.598
**Co**	0.547	0.523	0.261	1	0.928***	0.491	0.595	0.463	0.404	0.754*
**Ni**	0.428	0.428	0.261	0.928***	1	0.491	0.571	0.463	0.357	0.670
**Hg**	0.694	0.670	0.742*	0.491	0.491	1	0.467	0.672	0.287	0.626
**Cu**	0.785*	0.619	0.571	0.595	0.571	0.467	1	0.354	0.142	0.946***
**As**	0.327	0.545	0.709*	0.463	0.463	0.672	0.354	1	0	0.466
**Sr**	0.119	0.642	0.333	0.404	0.357	0.287	0.142	0	1	0.215
**Cr**	0.850**	0.706	0.598	0.754*	0.670	0.626	0.946***	0.466	0.215	1

Asterisks indicate a correlations are significant at *$P < 0.05$; **$P < 0.01$; ***$P < 0.001$).

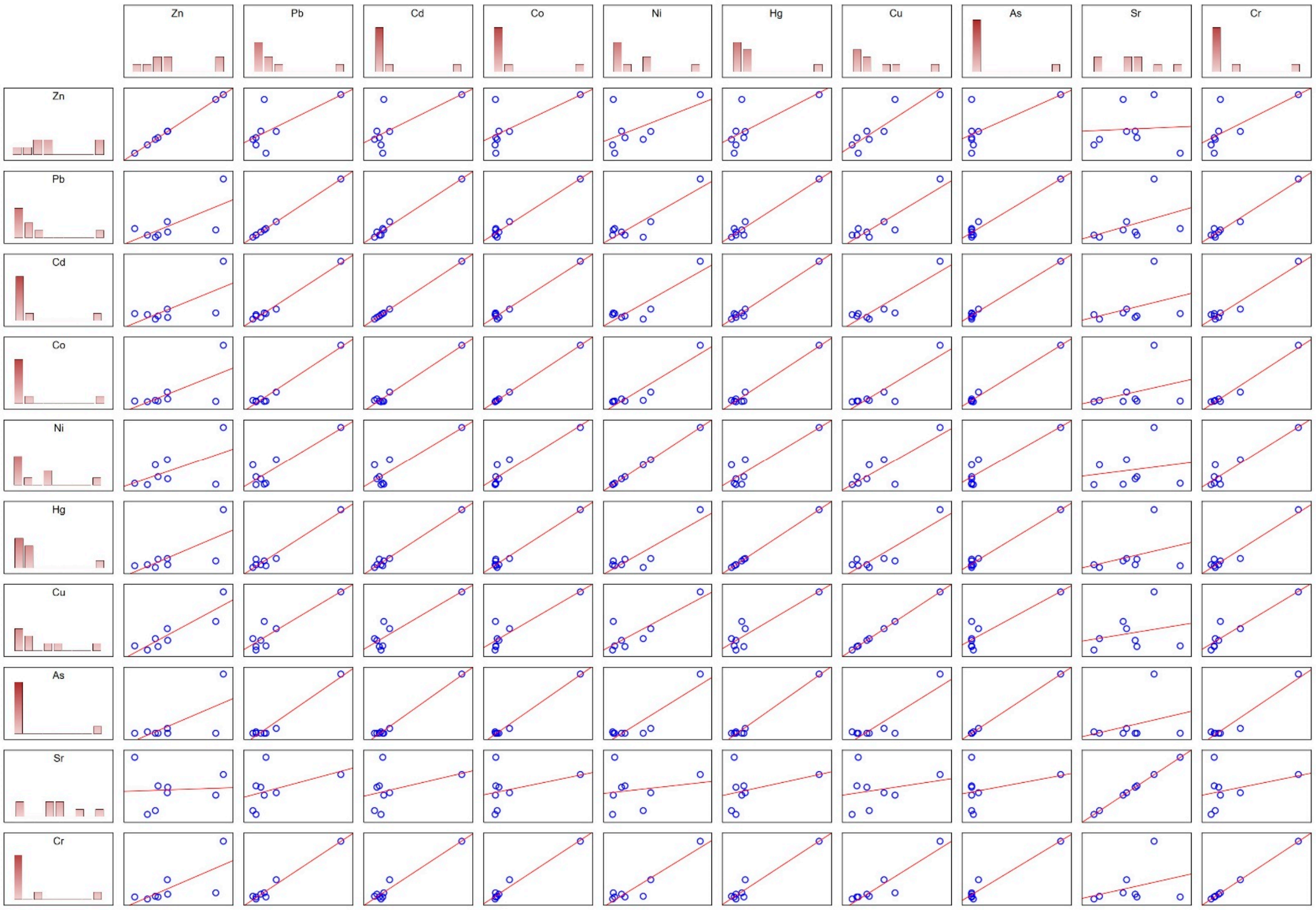

Supplement: Supplementary file 1 [file ijerph-19-09167-s001.zip › Table S6.pdf]
